# Comparative Analysis of the Gut Microbiota Composition between Captive and Wild Forest Musk Deer

**DOI:** 10.3389/fmicb.2017.01705

**Published:** 2017-09-05

**Authors:** Yimeng Li, Xiaolong Hu, Shuang Yang, Juntong Zhou, Tianxiang Zhang, Lei Qi, Xiaoning Sun, Mengyuan Fan, Shanghua Xu, Muha Cha, Meishan Zhang, Shaobi Lin, Shuqiang Liu, Defu Hu

**Affiliations:** ^1^College of Nature Conservation, Beijing Forestry University Beijing, China; ^2^College of Animal Science and Technology, Jiangxi Agricultural University Nanchang, China; ^3^Research Department, Zhangzhou Pien Tze Huang Pharmaceutical Co., Ltd. Zhangzhou, China

**Keywords:** gut microbiota, symbioses, functional analysis, Firmicutes, Bacteroidetes, forest musk deer

## Abstract

The large and complex gut microbiota in animals has profound effects on feed utilization and metabolism. Currently, gastrointestinal diseases due to dysregulated gut microbiota are considered important factors that limit growth of the captive forest musk deer population. Compared with captive forest musk deer, wild forest musk deer have a wider feeding range with no dietary limitations, and their gut microbiota are in a relatively natural state. However, no reports have compared the gut microbiota between wild and captive forest musk deer. To gain insight into the composition of gut microbiota in forest musk deer under different food-source conditions, we employed high-throughput 16S rRNA sequencing technology to investigate differences in the gut microbiota occurring between captive and wild forest musk deer. Both captive and wild forest musk deer showed similar microbiota at the phylum level, which consisted mainly of Firmicutes and Bacteroidetes, although significant differences were found in their relative abundances between both groups. α-Diversity results showed that no significant differences occurred in the microbiota between both groups, while β-diversity results showed that significant differences did occur in their microbiota compositions. In summary, our results provide important information for improving feed preparation for captive forest musk deer and implementing projects where captive forest musk deer are released into the wild.

## Introduction

The forest musk deer (*Moschus berezovskii*) belongs to the Moschidae family (Deng et al., 2014) and is a small ruminant unique to Asia. The musk secreted by adult forest musk deer is widely used in Asian traditional medicine and in the modern perfume industry (Meng et al., 2011). Forest musk deer used to be widely distributed in the mountains and forests of south and southwest China. However, the wild forest musk deer population has dropped sharply in the last 50 years, due to indiscriminate poaching and a reduction in natural habitat areas. In 2003, the forest musk deer has been listed as a national key protected species in China. To relieve resource pressure on wild forest musk deer, China has attempted artificial breeding of forest musk deer since the 1950s. After 60 years of research, some success has been achieved with captive breeding of forest musk deer populations in China. However, forest musk deer diseases remain a key factor obstructing the long-term development of forest musk deer breeding. At present, the common diseases of captive forest musk deer are gastrointestinal diseases, pneumonia, abscess disease, rumen impaction, parasitic diseases, urolithiasis, and so on (Xu et al., 2014). Among these diseases, gastrointestinal diseases are the most common and have high mortality rates, the mortality rates were about 30% (Yan et al., 2016). Gastrointestinal diseases often occur in alternate seasons in winter and spring, autumn and winter. As a result of the captive forest musk deer drinking unclean water, eating moldy feed or breeders poor processing of concentrated feed, changing the feed too fast. In the early stage of the gastrointestinal disease, feces shaped like porridge and when the disease is serious, the feces are watery (Zhu et al., 2012). Due to the above reasons, gastrointestinal diseases are always associated with dysregulation in the gut microbiota of forest musk deer (Zhou et al., 2016), when normal microbiota is affected by host or environment, their type, amount and activity were abnormal, alien bacteria are easily to invade, thus host digestion and metabolism will be affected. Forest musk deer are browsers, i.e., classical concentrate selectors (Hofmann, 1989). Wild forest musk deer feed on the leaves of a great variety of high-fiber plant species (Hu and Zhang, 1989), while captive forest musk deer feed on leaves and high protein and polysaccharide concentrates given by artificial feeding, and substantial differences exist between these two feeding methods. This could affect the composition and function of gut microbiota in forest musk deer. However, to date, there has been a lack of studies comparing the gut microbiota between wild and captive forest musk deer.

Animal gut microbiota began to colonize after birth and is functionally indispensable for maintaining the health of hosts. Together, the host and gut microbiota constitute intestinal microbiome that are involved in the correct functioning in the host immune and digestive systems. Various diseases can emerge once “host-gut microbiota” systems are unbalanced (Sjögren et al., 2009; El Aidy et al., 2015). The composition of the gut microbiota is not fixed and unchanging, as the age of the host, dietary composition, lifestyle and environment, and other factors all affect the composition of gut microbiota in the host (van der Wielen et al., 2000; Apajalahti et al., 2001; Hill et al., 2005). Data from previous studies have shown that diet is an important factor affecting the composition of the gut microbiota (Bäckhed et al., 2005; Turnbaugh et al., 2009). Studies with herbivorous have demonstrated that the gut microbiota can decompose cellulose and metabolically degrade food and toxins (Flint et al., 2008; Arumugam et al., 2011; Kohl et al., 2011). In addition, the presence of the gut microbiota can promote the differentiation of intestinal epithelial cells and resist to invasion by pathogenic microorganisms (Carding et al., 2015). Forest musk deer are ruminants, and comparing the gut microbiota between wild and captive forest musk deer may reveal the effects of dietary factors on the physiological functions of the digestive tract.

Microorganisms present in animal fecal matter reflects the overall composition of the gut microbiotal community (Eckburg et al., 2005). Studying fecal matter is also a non-invasive method that is facilitates easy sample collection (Durbán et al., 2011). The previous work done by Hu et al. (2017) used alpine and forest musk deer fecal samples showed that the bacterial diversity and within group similarity increased with age, as well as different composition and abundance of microbiota between musk deer species. By comparison, in this study, we collected fresh fecal matters from wild and captive forest musk deer in the same period. This was the first time to compare differences in the compositions of gut microbiota between wild and captive forest musk deer under different food-source conditions. The results of this research will provide a scientific basis for diagnosing diseases of the digestive system in forest musk deer and for improving feeding methods.

## Materials and Methods

### Animals and Sample Collection

Ten healthy adult forest musk deer of similar age (3.5–5.5 years old) and body size were selected from the Pien Tze Huang Forest Musk Deer Breeding Center in Baoji City, Shaanxi province. These individuals were born and raised in captivity and there are at least 30 generations due to musk deer breeding began at the end of 1950s. Ear tags were used to differentiate each individual forest musk deer. The musk deer were separated at night to allow for feces to be collected from specific individuals. On the evening of the first day of the experiment, the deer enclosure was thoroughly cleaned and fresh fecal samples were collected at dawn on the second day. Collection of fecal samples from wild forest musk deer was performed at the Tangjiahe National Nature Reserve in the Sichuan province. The feed of captive forest musk deer consisted mainly of fresh leaves, such as Spanish moss (*Usnea diffracta*), *Swida bretschneideri*, *Fraxinus chinensis*, *Acer mono*, and *Clematis armandii*, combined with foods having high protein and polysaccharide contents (such as soybeans and corn flour). In contrast, wild forest musk deer mainly consumed wild high-fiber plant leaves in their diets. Totally, nine wild forest musk deer feces samples were selected. Wild feces collection should meet the following two requirements: Firstly, Fresh and relatively large feces were collected to ensure that all collected samples were from adult forest musk deer. Secondly, owing to the forest musk deer is a solitary animal and have strongly territoriality behavior; also, according to the research, the home range of forest musk deer roughly between 2.8 and 7 hm^2^ and spread along the hillside (Wu and Wang, 2006). Thus, we took samples along the valley and select only one sample for each valley. The linear distance between the two valleys is at least 500 m, far beyond the maximum overlap between the forest musk deer home range. Therefore, we can ensure that these wild samples were all from different individuals. Sterile disposable gloves were used during sample collection to avoid human contamination. After sample collection, the samples were stored in sterile centrifuge tubes and sealed to avoid sample cross-contamination. All fresh fecal samples were immediately stored in liquid nitrogen for transportation back to the laboratory. Subsequently, samples were stored at -80°C until DNA extraction. It should be mentioned that all the fecal samples used in this experiment is quite different from Hu et al.’s (2017) research.

This study was carried out in accordance with the recommendations of the Institution of Animal Care and the Ethics Committee of Beijing Forestry University. The protocol was approved by the Ethics Committee of Beijing Forestry University. The collection of the captive musk deer stool samples was approved by the Pien Tze Huang Forest Musk Deer Breeding Center.

### DNA Extraction and Purification

Total bacterial DNA was extracted with the QIAamp DNA Stool Mini Kit (QIAGEN, Hilden, Germany) according to the manufacturer’s protocol. The integrity of the nucleic acids were determined visually by electrophoresis on a 1.0% agarose gel containing ethidium bromide. The concentration and purity of each DNA extract were determined using a Qubit dsDNA HS Assay Kit (Life Technologies, Carlsbad, CA, United States). The extracted total DNA was preserved at -80°C.

### MetaVx^TM^ Library Preparation and Illumina MiSeq Sequencing

Next generation sequencing library preparations and Illumina MiSeq sequencing were conducted at GENEWIZ, Inc. (Suzhou, China). DNA samples were quantified using a Qubit 2.0 Fluorometer (Invitrogen, Carlsbad, CA, United States). The 30–50 ng DNA was used to generate amplicons using a MetaVx^TM^ Library Preparation kit (GENEWIZ, Inc., South Plainfield, NJ, United States). V3, V4, and V5 hypervariable regions of microbial 16S rDNA were selected for generating amplicons and following taxonomy analysis. GENEWIZ designed a panel of proprietary primers aimed at relatively conserved regions bordering the V3, V4, and V5 hypervariable regions of the bacterial and archaeal 16S rRNA gene. (For samples containing eukaryotic DNA, only V3 and V4 regions will be amplified.) The V3 and V4 regions were amplified using forward primers containing the sequence “CCTACGGRRBGCASCAGKVRVGAAT” and reverse primers containing the sequence “GGACTACNVGGGTWTCTAATCC.” The V4 and V5 regions were amplified using forward primers containing the sequence “GTGYCAGCMGCCGCGGTAA” and reverse primers containing the sequence “CTTGTGCGGKCCCCCGYCAATTC.” The first round PCR, respectively, amplified the V3–V4 and V4–V5 regions to obtain the target fragment and part of the adapters sequence, and the second round PCR mixed the first round PCR amplification products. At the same time, indexed adapters were added to the ends of the 16S rDNA amplicons to generate indexed libraries ready for downstream NGS sequencing on Illumina Miseq. DNA libraries were validated by Agilent 2100 Bioanalyzer (Agilent Technologies, Palo Alto, CA, United States), and quantified by Qubit 2.0 Fluorometer. DNA libraries were multiplexed and loaded on an Illumina MiSeq instrument according to manufacturer’s instructions (Illumina, San Diego, CA, United States). Sequencing was performed using a 2×300/250 paired-end (PE) configuration; image analysis and base calling were conducted by the MiSeq Control Software (MCS) embedded in the MiSeq instrument.

### Data Analysis

The QIIME data analysis package was used for 16S rRNA data analysis. The forward and reverse reads were joined and assigned to samples based on barcode and truncated by cutting off the barcode and primer sequence. Quality filtering on joined sequences was performed and sequence which did not fulfill the following criteria were discarded: sequence length >200 bp, no ambiguous bases, mean quality score ≥ 20. Then the sequences were compared with the reference database (RDP Gold database) using UCHIME algorithm to detect chimeric sequence, and then the chimeric sequences were removed. The effective sequences were used in the final analysis. Sequences were grouped into operational taxonomic units (OTUs) using the clustering program VSEARCH (1.9.6) against the Silva 119 database pre-clustered at 97% sequence identity. The Ribosomal Database Project (RDP) classifier was used to assign taxonomic category to all OTUs at confidence threshold of 0.8. The RDP classifier uses the Silva 119 database which has taxonomic categories predicted to the species level. Novel clusters (OTUs that did not match the reference database) were removed when performing analysis.

Sequences were rarefied prior to calculation of alpha and beta diversity statistics. Alpha diversity indexes were calculated using the Mothur software (Schloss et al., 2009) from rarefied samples using for richness and diversity indices of bacterial community (i.e., ACE, Chao1, Shannon, and Simpson). Principal coordinate analysis (PCoA) performed using unweighted UniFrac. A one-way analysis of similarity (ANOSIM) was performed to determine the differences among groups (Clarke and Gorley, 2006). Here, the Bray–Curtis similarity index was used as a metric of similarity between the bacterial communities based on the abundance of OTUs between samples. The heatmap figures, Venn diagrams, and ANOSIM were produced using R^1^, and the cladogram was generated using the online LEfSe project^2^. Differences in phylum and genus relative abundances are presented as means ± SD. Student’s *t*-test was used for data analysis. A *P*-value < 0.05 was considered statistically significant. The raw sequences obtained in this study were available through the NCBI Sequence Read Archive (accession number SRR5839043).

## Results

### Analysis of rRNA Sequencing Results

**FIGURE 1:**
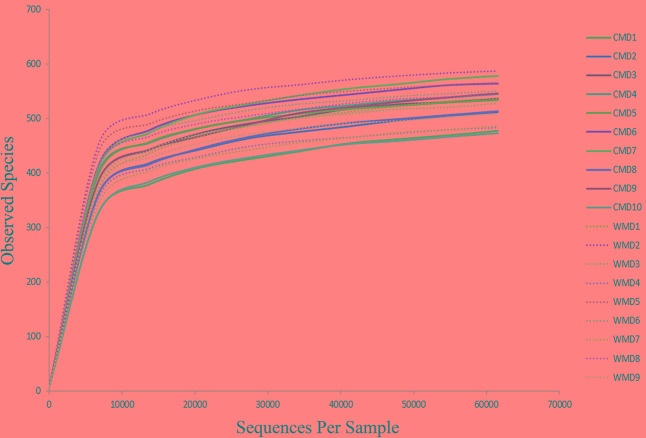
Rarefaction curves. The *x*-axis shows the number of valid sequences per sample and the *y*-axis shows the observed species (operational taxonomic units, OTUs). Each curve in the graph represents a different sample and is shown in a different color. As the sequencing depth increased, the number of OTUs also increased. Eventually the curves began to plateau, indicating that as the number of extracted sequences increased, the number of OTUs detected was decreased.

The Illumina MiSeq sequencing platform was used to amplify and detect 16S rRNA gene product sequences from fecal microbiota of 9 wild and 10 captive forest musk deer. After performing a series of processing steps with the sequencing results, 74,559–183,243 valid sequences were obtained from each sample, for a total of 2,156,408 sequences (average length of 444.28 base pairs). Supplementary Table 1 shows key aspects of the sequencing data from various samples after filtering. Supplementary Figure 1 shows the length distribution of valid sequences. A total of 813 OTUs were obtained at a sequence-similarity level of 97%. The rarefaction curves for the OTUs detected in this study showed that the quantity of observed species (OTUs) increased as the sequencing depth increased. The ends of the rarefaction curves taper off with increasing numbers of sequences per sample, as is commonly observed with sequencing data (**Figure 1**). Sequencing integrity was measured using Good’s coverage. The Good’s coverage value in our study approached 99%, showing that most bacterial species in our samples were detected. A ribosomal database was used to classify sequences representing OTUs. Detected bacteria were classified into 10 phyla, 20 classes, 32 orders, 50 families, and 98 genera.

### Comparison of the Gut Microbiota between Captive and Wild Forest Musk Deer

**FIGURE 2:**
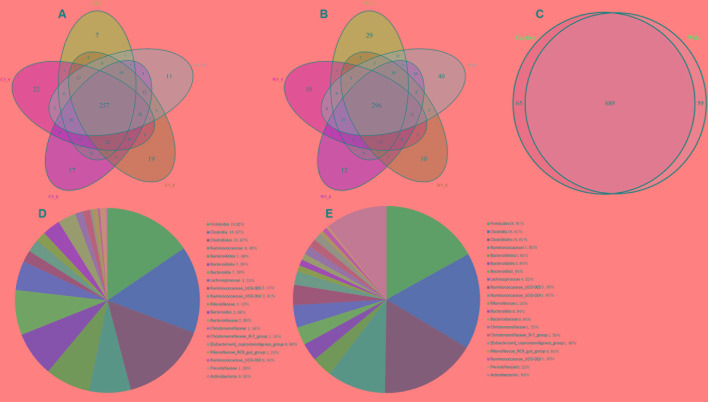
Venn diagram and pie charts. The Venn diagrams show the numbers of OTUs (97% sequence identity) that were shared or not shared by CMD and WMD individuals, respectively, depending of overlaps. For this presentation, two individuals had to be combined (e.g., C1_2) thereby reflecting the number of OTUs shared by both individuals. **(A)** The number of OTUs shared by CMD. **(B)** The number of OTUs shared by WMD. **(C)** The number of OTUs shared by CMD and WMD. The pie diagram shows the 20 most abundant taxa (calculated over the combined dataset) in CMD and WMD. **(D)** CMD, **(E)** WMD.

A Venn diagram was used to confirm the core gut microbiota present in wild and captive forest musk deer. Here, the shared taxa by all individuals in each group were deemed to be core bacterial communities. In the captive forest musk deer group, 257 OTUs common to all forest musk deer individuals were identified. In the wild forest musk deer group, the number of OTUs common to all forest musk deer individuals was 296. In addition, there are 689 OTUs shared by CMD and WMD. **Figures 2A–C** show the Venn diagrams. **Figures 2D,E** shows the 20 most abundant taxa (calculated over the combined dataset) in CMD and WMD.

### Diversity Analysis of Microbiota in Captive and Wild Forest Musk Deer

**FIGURE 3:**
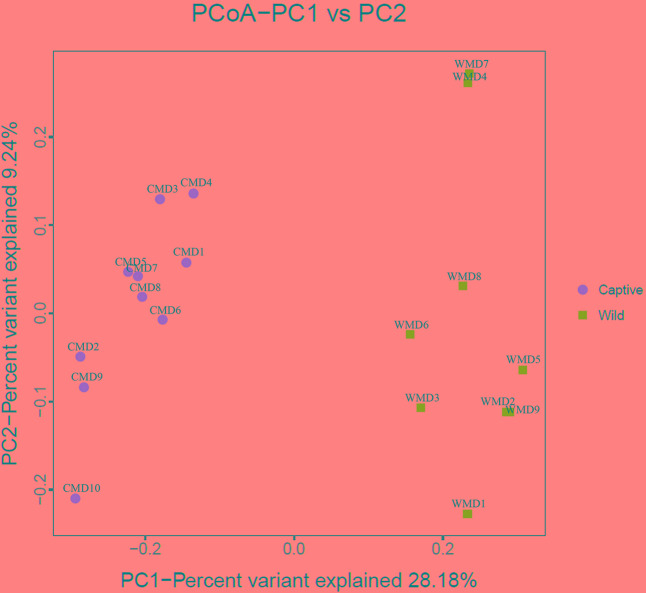
Principal coordinate analysis (PCoA) plot. Red dots represent captive forest musk deer samples, and blue squares represent wild forest musk deer samples. Samples in the same group are represented by the same color and shape. PC1_vs_PC2 is the PCoA plot obtained from the first and second main coordinates; the *x*-axis and *y*-axis represent the first and second main coordinates, respectively. The percentage of the main coordinates represent the relative contribution of this coordinate to sample differences, which is a measure of the amount of original information extracted by this main coordinate. The distances between the sample points represent the similarity of microbiota in the samples. A closer distance represents higher similarity and samples that cluster together are composed of similar microbiota.

We calculated α-diversity (Ace, Chao 1, Shannon, and Simpson) and β-diversity indices of captive and wild forest musk deer (**Table 1**). The Ace values in captive and wild forest musk deer were 569.27 ± 35.37 and 557.89 ± 31.92, respectively (*P* > 0.05). The Chao1 values in the CMD and WMD groups were 579.02 ± 35.41 and 563.94 ± 29.79, respectively (*P* > 0.05). The Shannon indices in the CMD and WMD groups were 6.85 ± 0.24 and 6.88 ± 0.45, respectively (*P* > 0.05). The Simpson indices in the CMD and WMD groups were 0.98 ± 0.01 and 0.98 ± 0.02, respectively (*P* > 0.05). No significant differences were observed in the α-diversity indices of the CMD and WMD groups (*P* > 0.05), while the β-diversity results did reveal significant differences between both groups. PCoA demonstrated the differences of microbiota in the captive and wild samples (**Figure 3**), the ordination plot shows that the captive and wild samples are clearly separated, which means that the microbial communities in captive and wild samples have great differences.

**Table 1:** Comparison of α-diversity indices of gut microbiota from the CMD and WMD groups.

Alpha diversity	CMD	WMD	*P*-value
Ace	569.27 ± 35.37	557.89 ± 31.92	*P* = 0.47
Chao 1	579.02 ± 35.41	563.94 ± 29.79	*P* = 0.33
Shannon	6.85 ± 0.24	6.88 ± 0.45	*P* = 0.88
Simpson	0.98 ± 0.01	0.98 ± 0.02	*P* = 0.59
Observed species (OTUs)	528.14 ± 34.33	532.50 ± 34.91	*P* = 0.79


*The Ace and Chao index was used to estimate the number of operational taxonomic units (OTUs) in samples and is commonly used in ecology to estimate the total number of species. The Shannon index and Simpson’s diversity index are common measures of diversity, which reflect richness and evenness of the samples.*

### Analysis of Differences between the CMD and WMD Gut Microbiota

**FIGURE 4:**
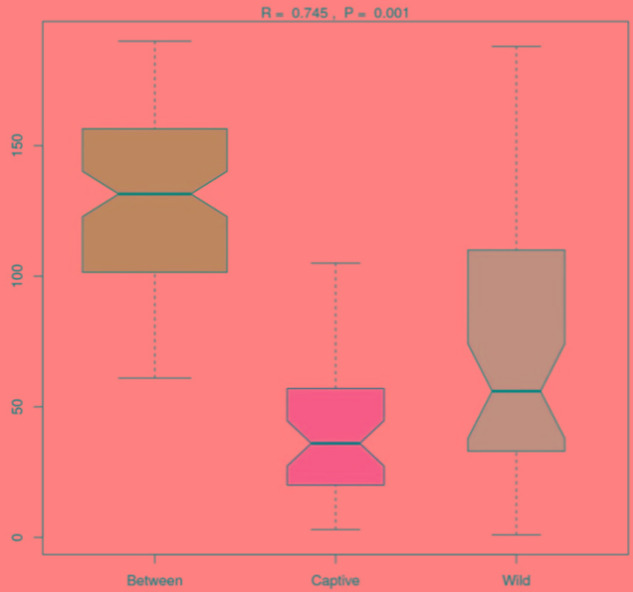
ANOSIM analysis. *R*-value: *R*-value range (–1, 1). Actual results are generally between 0 and 1. An *R*-value close to 0 represents no significant inter-group and intra-group differences. A *R*-value close to 1 shows that inter-group differences are greater than intra-group differences. *P*-value: The *P*-value represents the confidence level of the statistical analysis; *P* < 0.05 reflects a statistically significant difference. The *y*-axis represents the distance rank between samples, and the *x*-axis represents the results between both groups. Intra-group results are shown for each group. In the plot, the *R*-value was close to 1, indicating that inter-group differences were greater than the intra-group differences, and *P* < 0.05 shows that this result was statistically significant.

**FIGURE 5:**
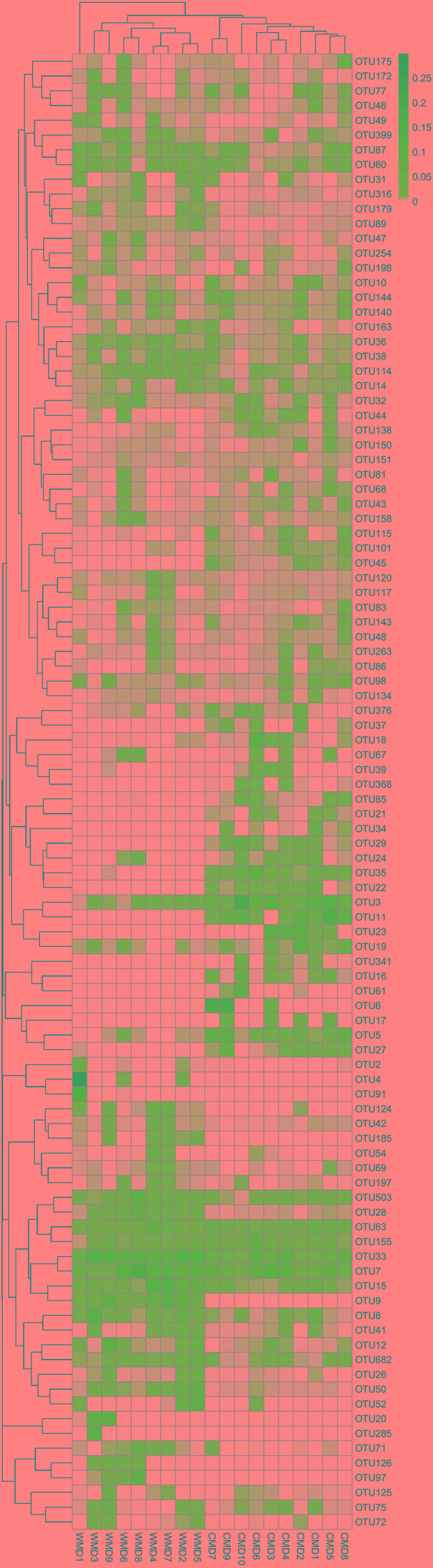
Heatmap of the 100 most abundant OTUs. Title for rows are OTU ID and title for columns are sample information. The left side of the graph is the OTU clustering tree and top is the sample cluster tree. Each color of the grid in the middle heatmap represents the value of the OTU relative abundance in each row after normalization processing.

**FIGURE 6:**
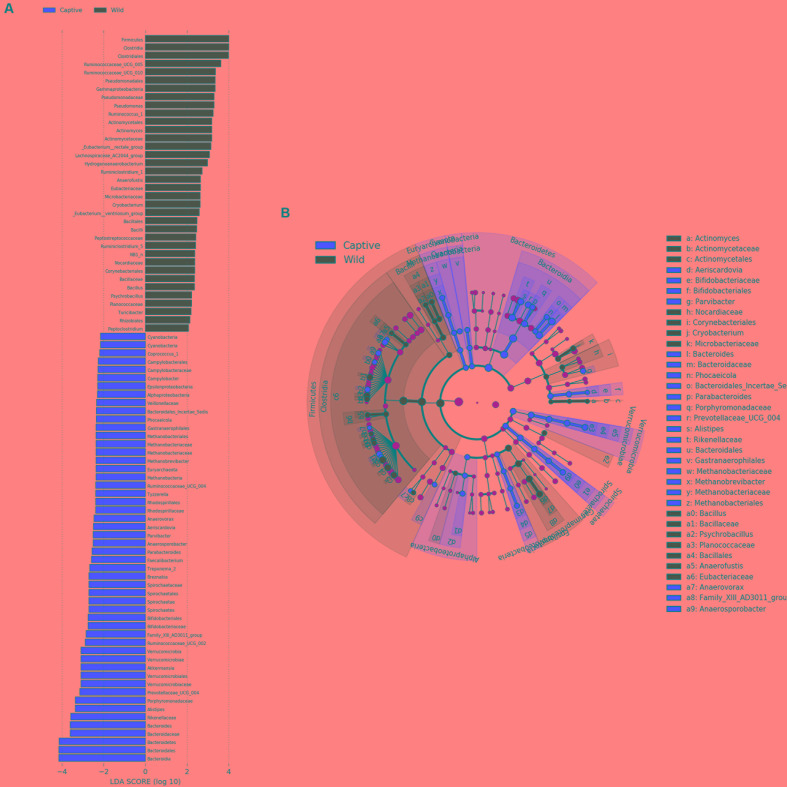
LEfSe analysis. **(A)** Plot from LEfSe analysis. The plot was generated using the online LEfSe project. The length of the bar column represents the LDA score. The figure shows the microbial taxa with significant differences between the CMD (red) and WMD (green) (LDA score > 2). **(B)** A cladogram showing the differences in relative abundance of taxa at five levels between CMD and WMD. The plot was generated using the online LEfSe project. The red and green circles mean that CMD and WMD showed differences in relative abundance and yellow circles mean non-significant differences.

Analysis of similarities (ANOSIM) demonstrated the difference in the gut microbiota between captive and wild forest musk deer (*R* = 0.745, *p* = 0.001; **Figure 4**). The inter-group differences in gut microbiota composition of CMD and WMD were greater than the intra-group differences, and the composition difference in the gut microbiota between both groups was significant (*P* < 0.05). The heatmap of the 100 most abundant OTUs show the similarities and differences between the CMD and WMD (**Figure 5**). Moreover, plot from LEfSe analysis (**Figure 6A**) display LDA scores of microbial taxa with significant differences between CMD and WMD, and the cladogram (**Figure 6B**) showed differences in 88 taxa between CMD and WMD.

**FIGURE 7:**
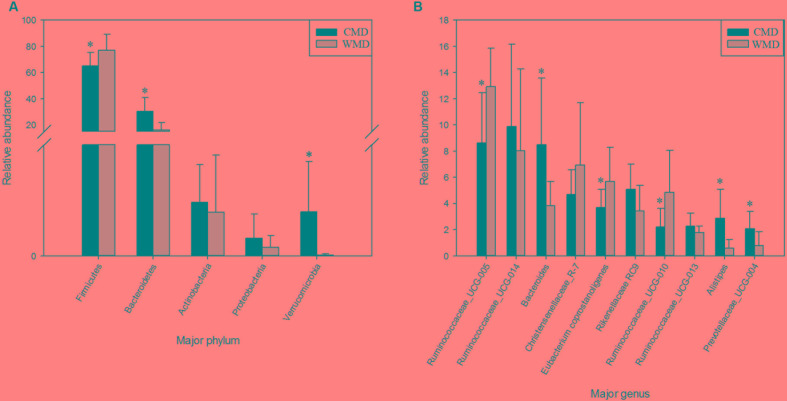
Differences in relative abundance (mean % ± SD) of 5 major bacterial phyla and 10 major bacterial genus between the CMD and WMD groups. **(A)** Relative abundance (mean % ± SD) of five major bacterial phyla between the CMD and WMD groups. **(B)** Relative abundance (mean % ± SD) of 10 major bacterial genus between the CMD and WMD groups. The significance of Firmicutes, Bacteroidetes, Actinobacteria, and Verrucomicrobia was determined using the independent-sample *t*-test, whereas the non-parametric Mann–Whitney *U* test was used to examine the significance of Proteobacteria. ^∗^*P* < 0.05.

**Figures 7A,B** shows the differences in relative abundances at the phylum level of the top 5 bacterial communities and genus level of the top 10 bacterial communities in captive and wild forest musk deer. In wild forest musk deer, the relative abundance of Firmicutes was significantly higher than that in captive forest musk deer (*P* < 0.05). In contrast, the relative abundances of the Bacteroidetes and Verrucomicrobia phyla in wild forest musk deer were significantly lower in captive forest musk deer (*P* < 0.05). Both groups did not show any significant differences in the relative abundances of Actinobacteria and Proteobacteria (*P* > 0.05). At the genus level, the relative abundances of Ruminococcaceae UCG-005, *Eubacterium coprostanoligenes* and Ruminococcaceae UCG-010 in wild forest musk deer were significantly higher compared with captive forest musk deer (*P* < 0.05), while the relative abundance of Bacteroides, Alistipes, and Prevotellaceae_UCG-004 was lower in wild forest musk deer than in captive forest musk deer (*P* < 0.05). No significant differences were found in Ruminococcaceae UCG_014, Christensenellaceae_R-7, Rikenellaceae RC9, and Ruminococcaceae UCG-013 (*P* > 0.05) between both groups.

## Discussion

This study is the first to employ 16S rRNA Illumina MiSeq high-throughput sequencing technology to compare differences in the gut microbiota between captive and wild forest musk deer. The gut microbial ecosystem is an evolving system, and its species diversity and abundance play important functional roles in maintaining normal physiology in the host. However, the gut microbiota is also affected by the host (Koboziev et al., 2014). Data from previous local and overseas studies have shown that diet is the main factor affecting the gut microbiota in mammals (Ley et al., 2008; Schwab et al., 2011; Navarrete et al., 2012). In this study, the feed of captive forest musk deer consisted mainly of fresh leaves combined with foods having high protein and polysaccharide contents. While, wild forest musk deer mainly consumed wild high-fiber plant leaves. Thus, differences of microbiota between captive and wild musk deer might be strongly associated with the dietary differences.

The results of this study revealed that the core microbiota in captive and wild forest musk deer are mainly classified into the Firmicutes and Bacteroidetes phyla (**Figure 7A**). This result was consistent with previous studies of the gut microbiota in ruminants (Sundset et al., 2007; Gruninger et al., 2014; Ishaq and Wright, 2014). For example, Koike et al. (2003) studied ruminal bacteria diversity in sheep and found that the proportion of Firmicutes and Bacteroidetes accounted for >80% of the total ruminal bacteria. In this study, we found that the abundance of Firmicutes in the gut of wild forest musk deer was significantly higher than in captive forest musk deer. However, the abundance of Bacteroidetes in the gut of captive forest musk deer was significantly higher than in wild forest musk deer. Firmicutes are the main cellulolytic bacteria, and they can degrade cellulose into volatile fatty acids for the host to use. The main function of Bacteroides is to help the host degrade carbohydrates (especially polysaccharides), proteins, and other substances to increase the nutrient-utilization rate of the host (Bäckhed et al., 2004). Bacteroides can also promote immune system development to enhance host immunity (Hooper, 2004), and maintain intestinal microbial ecological balance (Hooper et al., 2001; Sears, 2005). This finding indicated that intestinal microorganisms also show adaptive changes under long-term human captive breeding (Fernando et al., 2010). At the same time, Bacteroides are opportunistic pathogens. When the normal microbial ecological balance is disrupted, these bacteria can cause endogenous infections. Previous findings have shown significant differences in the gut microbiota in animals with gastrointestinal diseases and healthy individuals: some Bacteroides species were increased while lactic acid bacteria species were decreased (Marteau et al., 2004). The high incidence of gastrointestinal diseases in captive forest musk deer could be caused from such conditions. In addition, similar core bacterial species between captive and wild forest musk deer at the genus level. Among these species, the relative abundance of *Ruminococcus* was at a relatively high level, and the amount of *Ruminococcus* in wild forest musk deer was significantly higher than in captive forest musk deer. *Ruminococcus* also plays a crucial role in dietary fiber digestion in ruminants (Han et al., 2015). This result was consistent with the differences in feeding habits observed between wild and captive forest musk deer. In addition, In **Figures 5**, **6**, we can see that almost all captive musk deer have *Akkermansia* and *Bifidobacterium*, which are mostly absent from wild musk deer. While, *Akkermansia* and *Bifidobacterium* are common in humans but not found in all other mammals, this situation may due to more contact with humans under captive conditions. Furthermore, some captive musk deer contain potential pathogens like *Campylobacter* and *Escherichia*, which may cause gastrointestinal diseases such as diarrhea. At present, captive musk deer always have high incidence of intestinal diseases, this is likely to be affected by human beings and deserve further study.

The α-diversity and gut microbiota composition did not show significant differences in captive and wild forest musk deer. This was not consistent with the results of Hu et al. (2017), who studied gut microbiota in alpine musk deer and forest musk deer. In addition to its being affected by diet, the gut microbiota diversity was also associated with factors such as the host genotype and age (Kovács et al., 1972). In this study, the captive and wild forest musk deer were genetically similar, and the relative abundances of various microbiota may only have been affected by food, while the main microbiota composition was unchanged. Beyond this consideration, seasonal factors may also cause no significant differences in diversity. In this study, approximately 35.70% of the sequences were not classified to any known genera, suggesting that there could be new, unknown bacteria species present in the gut of forest musk deer. These microbiotas require further study.

## Conclusion

This study showed that the diet affects the gut microbiota of forest musk deer to some extent. Feed is the material basis for breeding musk deer. Under captive breeding conditions, all feed consumed during the entire life of a musk deer is totally dependent on the supply by humans. Different feed combinations have differing degrees of impact on the growth, reproduction, health, and disease susceptibility of musk deer. For forest musk deer that live in the wild and can feed freely, their gut microbiotas have been formed through hundreds of years of long-term evolutionary processes and are in a relatively normal state. In contrast, captive forest musk deer only have a few decades of history. This form of artificial changes to the diet of forest musk deer will inevitably affect the physiological immunity of forest musk deer. The high-protein and high-polysaccharide content feed currently given to captive forest musk deer has resulted in greater levels of starch fermentation in the digestive systems of forest musk deer and the production of large quantities of acidic substances (Le et al., 2007). This, in turn, causes the gastrointestinal tract to be in a chronic acidic environment and possibly causes some damage to the digestive tract. Currently, whether the high incidence of abscesses in captive forest musk deer is due to the above reasons requires further in-depth study. Therefore, forest musk deer breeding centers could change the composition of gut microbiota in captive forest musk deer by appropriate increases in high-fiber plant leaves and appropriate decreases in the proportion of concentrate feed. As far as possible, this will cause the gut microbiota in captive forest musk deer to return to a natural state similar to that observed in wild forest musk deer. This is significant for improving the health of captive forest musk deer and implementation of projects where captive forest musk deer are released into the wild. Nevertheless, the detailed functional analysis are still limited and needed to have further research. Further studies are essential to determine the contributions of the bacterial taxa and metabolic pathways to the immunology and physiology of forest musk deer.

## Author Contributions

YL, SqL, and DH conceived and designed the experiments. YL, XH, SY, and JZ carried out the DNA extraction and data analysis. MF, XS, SX, and MC participated in the sample collection. YL, XH, and TZ wrote the paper. LQ, MZ, and SbL assist with experiments and advice on manuscript content. All authors read and approved the final manuscript.

## Conflict of Interest Statement

The authors declare that the research was conducted in the absence of any commercial or financial relationships that could be construed as a potential conflict of interest.

## Footnotes

1 http://www.r-project.org/

2 https://huttenhower.sph.harvard.edu/galaxy/
