# Supplementary material for: Comparative Analysis of the Gut Microbiota Composition between Captive and Wild Forest Musk Deer
Source: Front Microbiol. 2017 Sep 5;8:1705. doi: 10.3389/fmicb.2017.01705 (PMC5591822; doi:10.3389/fmicb.2017.01705)
Supplement: Supplementary file 1 [file Data_Sheet_1.docx]

Supplementary Material

**Comparative analysis of the gut microbiota composition between captive and wild forest musk deer**

**Yimeng Li ^1^**^*^**, Xiaolong Hu ^1, 2^**^*^**, Shuang Yang^1^, Juntong Zhou^1^, Tianxiang Zhang^1^, Lei Qi^1^, Xiaoning Sun^1^, Mengyuan Fan^1^, Shanghua Xu^1^, Muha Cha^1^, Meishan Zhang^1^, Shaobi Lin ^3^, Shuqiang Liu*^,1, 3^, Defu Hu*^,1^**

*****Corresponding authors: Shuqiang Liu, [liushuqiang@bjfu.edu.cn](mailto:liushuqiang@bjfu.edu.cn); Defu Hu, hudf@bjfu.edu.cn

1. Supplementary data

2. Supplementary Figures and Tables

| Sample | PE_reads | Nochimera | AvgLen (bp) | GC (%) | Effective (%) |
| --- | --- | --- | --- | --- | --- |
| CMD1 | 109260 | 102419 | 445.74 | 52.08 | 93.74 |
| CMD2 | 114162 | 107433 | 444.32 | 52.22 | 94.11 |
| CMD3 | 138558 | 126916 | 444.82 | 52.72 | 91.6 |
| CMD4 | 123087 | 113464 | 443.45 | 52.92 | 92.18 |
| CMD5 | 106655 | 98196 | 445.65 | 52.78 | 92.07 |
| CMD6 | 108439 | 97517 | 445.37 | 52.5 | 89.93 |
| CMD7 | 123999 | 114248 | 444.19 | 53.16 | 92.14 |
| CMD8 | 111307 | 105380 | 442.47 | 52.63 | 94.68 |
| CMD9 | 197039 | 183243 | 445.71 | 52.78 | 93 |
| CMD10 | 121747 | 111464 | 449.92 | 51.39 | 91.55 |
| WMD1 | 133956 | 122028 | 447.99 | 53.59 | 91.1 |
| WMD2 | 133118 | 121949 | 442.97 | 52.79 | 91.61 |
| WMD3 | 117777 | 111442 | 443.08 | 52.3 | 94.62 |
| WMD4 | 110019 | 104404 | 441.69 | 53.56 | 94.9 |
| WMD5 | 139906 | 129646 | 442.81 | 52.85 | 92.67 |
| WMD6 | 121794 | 115564 | 441.89 | 53 | 94.88 |
| WMD7 | 78434 | 74559 | 442.89 | 53.32 | 95.06 |
| WMD8 | 110782 | 101078 | 442.55 | 53.14 | 91.24 |
| WMD9 | 124182 | 115458 | 443.76 | 52.8 | 92.97 |

**Supplementary Table 1. Statistical table of post-filtering sequencing data.** Sample: Name of sequencing sample (CMD: captive musk deer; WMD: wild musk deer); Paired-End (PE) reads: Number of original PE reads; No chimera: Number of valid sequences after removal of chimeras; AveLen (bp): Average length of valid sequences; GC (%), GC content of valid data; Effective (%): The percentage of valid sequences after chimera removal over the original number of PE reads.


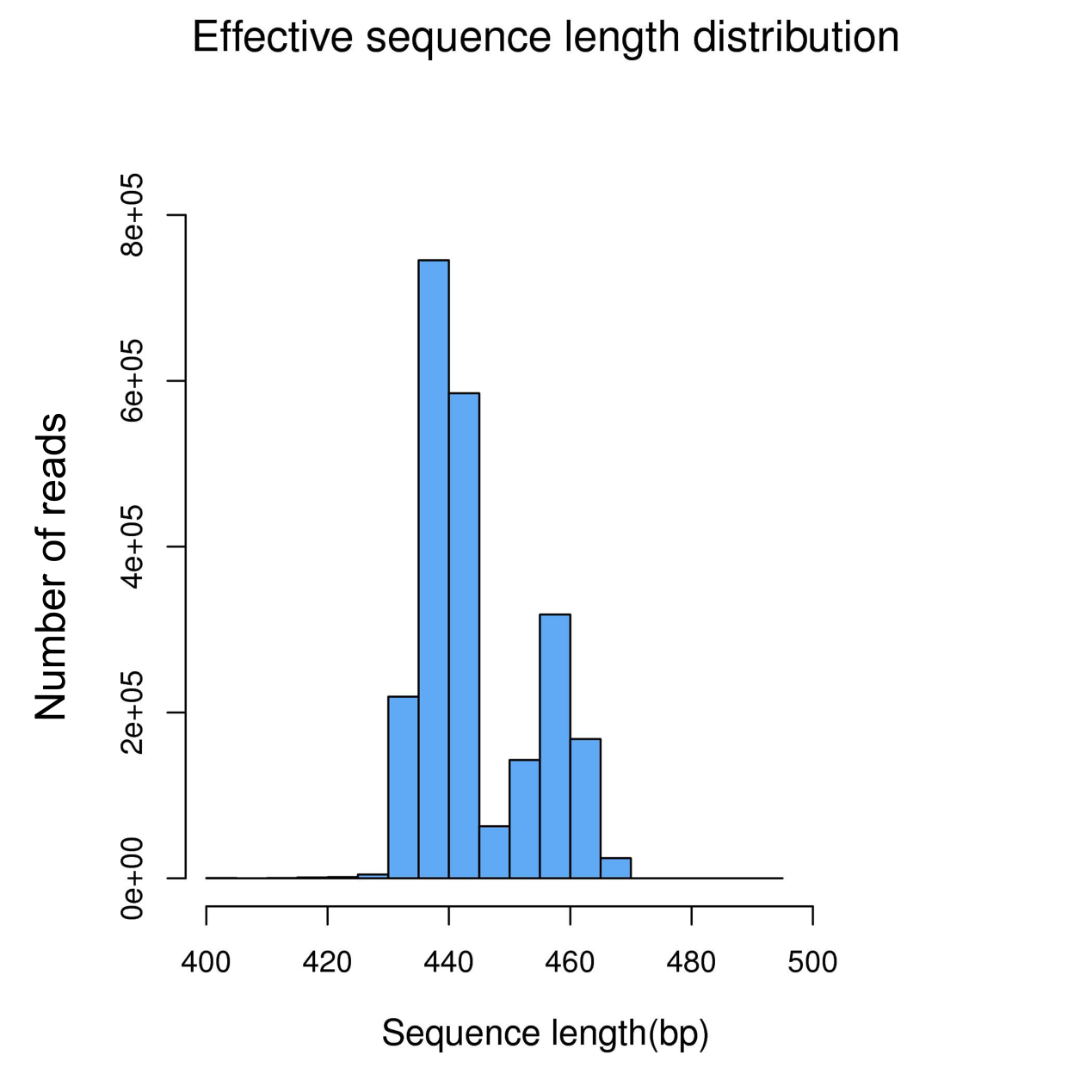


**Supplementary Figure 1. Effective sequence-length distribution.** The X-axis shows the sequence length (bp) and the Y-axis shows the number of reads with each different length (Number of reads).
